# Prediction of Early TBI Mortality Using a Machine Learning Approach in a LMIC Population

**DOI:** 10.3389/fneur.2019.01366

**Published:** 2020-01-24

**Authors:** Robson Luis Amorim, Louise Makarem Oliveira, Luis Marcelo Malbouisson, Marcia Mitie Nagumo, Marcela Simoes, Leandro Miranda, Edson Bor-Seng-Shu, Andre Beer-Furlan, Almir Ferreira De Andrade, Andres M. Rubiano, Manoel Jacobsen Teixeira, Angelos G. Kolias, Wellingson Silva Paiva

**Affiliations:** ^1^School of Medicine, Federal University of Amazonas (UFAM), Manaus, Brazil; ^2^Division of Neurosurgery, Hospital das Clinicas, University of São Paulo, São Paulo, Brazil; ^3^Department of Anesthesiology, Hospital das Clinicas, University of São Paulo, São Paulo, Brazil; ^4^Anhembi Morumbi Univesity, São Paulo, Brazil; ^5^Department of Neurosurgery, Wexner Medical Center, Ohio State University, Columbus, OH, United States; ^6^Neurosciences Institute, El Bosque University, Bogota, Colombia; ^7^NIHR Global Health Research Group on Neurotrauma, University of Cambridge, Cambridge, United Kingdom

**Keywords:** prognostic, traumatic brain injury, machine learning, mortality, LMICs

## Abstract

**Background:** In a time when the incidence of severe traumatic brain injury (TBI) is increasing in low- to middle-income countries (LMICs), it is important to understand the behavior of predictive variables in an LMIC's population. There are few previous attempts to generate prediction models for TBI outcomes from local data in LMICs. Our study aim is to design and compare a series of predictive models for mortality on a new cohort in TBI patients in Brazil using Machine Learning.

**Methods:** A prospective registry was set in São Paulo, Brazil, enrolling all patients with a diagnosis of TBI that require admission to the intensive care unit. We evaluated the following predictors: gender, age, pupil reactivity at admission, Glasgow Coma Scale (GCS), presence of hypoxia and hypotension, computed tomography findings, trauma severity score, and laboratory results.

**Results:** Overall mortality at 14 days was 22.8%. Models had a high prediction performance, with the best prediction for overall mortality achieved through Naive Bayes (area under the curve = 0.906). The most significant predictors were the GCS at admission and prehospital GCS, age, and pupil reaction. When predicting the length of stay at the intensive care unit, the Conditional Inference Tree model had the best performance (root mean square error = 1.011), with the most important variable across all models being the GCS at scene.

**Conclusions:** Models for early mortality and hospital length of stay using Machine Learning can achieve high performance when based on registry data even in LMICs. These models have the potential to inform treatment decisions and counsel family members.

**Level of evidence:** This observational study provides a level IV evidence on prognosis after TBI.

## Background

Traumatic brain injury (TBI) is a significant healthcare and economic problem in low- and middle-income countries (LMICs) (1, 2), accounting for a major burden regarding morbidity, mortality, disability, socioeconomic losses, reduced life expectancy, and the quality of life (2, 3). Road traffic accidents account for over 60% of all traumatic brain injuries, followed by falls (30%) and violence (10%), with the most affected age group being between 21 and 30 years old (1, 2). Although being able to predict a patient's prognosis at presentation accurately is a significant step in clinical decision making and the assessment of the quality of care (4), to our knowledge there are few prediction models from LMICs that have used machine learning methods based on data directly captured from registries in these environments.

To date, most attempts to predict outcomes after TBI has relied on the use of manually calculated scores. As one of its primary examples, the Glasgow Coma Scale (GCS) has been widely used to predict outcomes, associating patients with a score of 13–14 with longer post-traumatic amnesia and a higher rate of abnormal brain image findings at 6 months after the initial trauma. This score is not perfect, however, with no significant differences noticed for neuropsychiatric status, psychological distress, the frequency of somatic complaints, and the rate of return to work, among other factors (5). The GCS also has the potential to present its predictive performance enhanced by the inclusion of other variables, as has been demonstrated in the improvement of prediction accuracy of hospital mortality (6) through the integration of variables such as age and brain stem reflexes (7). In another example, when the GCS was combined with the Injury Severity Score, their joint performance significantly improved in comparison with isolated scores or the Abbreviated Injury Score for outcomes measured 12 months after the initial injury (8). Recently, GCS prediction models have been enhanced with pupillary response (9). In summary, prediction models including multiple variables tend to exceed isolated, manually calculated scores (10), opening up an opportunity for the use of prediction models as they can increase predictive performance (11, 12).

About the validation of prediction models in precise patient populations, one size does not fit all. In previous systematic reviews, prediction models demonstrated a wide variation in accuracy across different populations (11–13). Accounting for this variability were factors such as model validation using small samples, poor modeling methodology, and the lack of validation using external populations. Of particular relevance in the context of LMICs, models drawn up with samples from developed countries presented a worse performance when applied in LMICs, likely due to the mismatch about case mix as well as overall healthcare infrastructure (14). Hence, these results call for the specific development of models for use within a particular local population. However, to achieve this, local registry data collection is required, which can be a significant challenge in countries with scarce resources.

In the face of this gap in the literature, the objective of this article is to evaluate the predictive performance of a machine-learning-based model for mortality and length of stay applied to a LMIC cohort of TBI patients. We based this model on a prospective registry of a tertiary hospital from São Paulo, Brazil.

## Methods

Our objective was to develop a machine learning predictive model based on a prospective registry with consecutively enrolled TBI patients in Brazil. We described our modeling strategy according to the TRIPOD statement recommendations for the reporting of prognostic models (15).

### Ethics

The Institutional Review Board of the University of São Paulo (São Paulo, Brazil) approved of our study (CAAE 46831315.3.0000.0068), and informed consent was offered to all potential participants and subsequently signed before the implementation of any study protocol.

### Setting

We collected data from consecutive patients coming to the main trauma hospital of the state of São Paulo, Brazil (Hospital das Clinicas, University of São Paulo Medical School). Participant data were captured between March of 2012 and January of 2015, with the end of follow-up occurring in June of 2015.

### Participants

Our registry includes patients with TBI, defined as any patient requiring admission to an intensive care trauma unit as referred by the neurosurgery team. Pre-hospital data were collected through the analysis of the clinical chart of the rescue team. We only included patients aged 14 years old and above and patients depicting intracranial abnormality on initial head computerized tomography (CT) scan. We excluded patients with penetrating TBI, as well as those with a GCS of 15 and not associated intracranial lesions on the CT scan. In our institution, any patient with intracranial abnormalities is eligible to be transferred to ICU, which is subject to the availability of bed. Therapeutic planning followed recommendations provided by the Advanced Trauma Life Support as well as guidelines by the Brain Trauma Foundation whenever possible. A total of 517 participants were part of this analysis.

### Outcomes

The primary outcome was death within 14 days. We choose this outcome since previous studies in our institution showed a great number of missing data with long-term outcomes in this population. A recent randomized control trial performed in Latin America (16) used the long-term outcome to evaluate the value of intracranial pressure monitoring (ICP) regarding prognosis. There was a trend of benefit in early mortality in those who received ICP monitoring; however, no differences were found in the long term. Maybe the scarce rehabilitation programs in LMICs may have offset the initial benefit found. As we decided to use a strong outcome, which could also be compared with another predicting model (e.g., CRASH prognostic model), we choose 14 day mortality. Those patients who had been discharged before 14 days were contacted by telephone or mail. Secondary outcomes were hospital mortality (defined as death occurring during the hospital stay), the number of days spent in the ICU, and the number of days spent in the hospital.

### Predictors

Predictors were selected based on previously described models from TBI literature (4, 11–13). Specifically, we selected gender, age, level of pupil reactivity at admission, GCS at the scene where the trauma occurred (prehospital GCS), GCS at admission, the motor component score of the GCS, and presence of hypoxia and hypotension. Also included were midline shift bigger than 5 mm, brain herniation detected on CT (defined as effacement of the third ventricle or the basal cisterns), subarachnoid hemorrhage, epidural hemorrhage, subdural hemorrhage, intracerebral hemorrhage, trauma severity, prothrombin time, and partial thromboplastin time. We chose a larger amount of variables in order to find any predictor not found in calculators such as CRASH or IMPACT since our study population was composed only of cases from a LMIC hospital. These variables were all collected at ICU admission.

### Data Analysis

We initially performed a graphical exploratory analysis evaluating the frequency, percentage, and near-zero variance for all categorical variables, distribution for numeric variables, and missing values and patterns of all variables (17). Also, a MINE algorithm (18) was run in an exploratory manner to guide bivariate plot inspection. We proceed then with feature engineering including variable transformations and dummy coding for variables with distributions that were not normal at inspection, variable re-categorization removal for near-zero variation, and different imputation algorithms for variables with missing values. We modeled outcomes and predictors in the described format. To train and test our models, we used a 5-fold model validation.

Machine learning classification models for the prediction of categorical variables included regularized least squares and linear regression. Machine learning regression models, i.e., those directed at numeric outcomes, included random forest, neural network, decision tree, boosting, generalized linear model, partial least squares, and multivariate adaptive regression splines. Regression models for the classification of numeric variables included random forest, discriminant analysis, Bayesian methods, neural network, decision tree, boosting, generalized linear model, partial least squares, and multivariate adaptive regression splines. Comparison across models was performed using the area under the curve, sensitivity, specificity, kappa values as well as positive and negative predictive values. Since the length of stay in hospital and ICU did not present a normal distribution, all models were run with log-transformed variables and then subsequently exponentiated so that results could be clinically interpretable.

All calculations were performed using the statistical language R and the packages ggplot2, caret, knitr, vcd, randomForest, MASS, glmnet, mda, pROC, corrplot, and tabplot.

## Results

Five hundred seventy patients were admitted to the ICU with diagnosis of TBI. We excluded 26 patients who were victims of gunshot wound, 14 patients who had chronic subdural hematomas, 9 patients with GCS of 15 and no abnormalities on CT scan, 2 patients aged <2 years old, and 2 patients transferred after more than 48 h of trauma. Table 1 reports information on our total study sample as well as stratification by TBI severity. Comparisons were performed using chi-square and t-tests. Most patients were male (85.1%) with a mean age of 41.5 ± 18.1 [standard deviation (SD)] years. The average length of stay was 25.4 (SD 28.9) days, with 13.1 (SD 15.9) days spent in the intensive care unit. We found overall mortality at 14 days of 22.8%, with mortality rates increasing proportionally to severity levels. Higher severity scores were also associated with increased rates of hemorrhage at the epidural, subdural, and subarachnoid levels, as well as with worse GC.

**Table 1:** Sample description stratified by TBI severity.

**Variables**	**Total (*N* = 517)**	**Missing (*N* = 35)**	**Severe (*N* = 310)**	**Moderate (*N* = 64)**	**Mild (*N* = 108)**	***p***
Age in years	41.5 ± 18.1	46.2 ± 19.1	38.0 ± 16.0	48.7 ± 19.8	45.7 ± 20.2	<0.001
Male	440 (85.1%)	30 (85.7%)	269 (86.8%)	50 (78.1%)	91 (84.3%)	0.36
Length of stay in days	25.4 ± 28.9	22.4 ± 19.5	28.4 ± 31.4	23.3 ± 29.0	19.3 ± 22.1	0.031
ICU stay in days	13.1 ± 15.9	12.8 ± 13.0	15.1 ± 17.5	11.8 ± 13.6	8.1 ± 12.0	0.001
Reactive pupils at admission						<0.001
One reactive	63 (12.9%)	5 (15.6%)	52 (17.7%)	3 (4.8%)	3 (3.0%)	
None reactive	29 (6.0%)	2 (6.2%)	23 (7.8%)	0 (0.0%)	4 (4.0%)	
Both reactive	395 (81.1%)	25 (78.1%)	218 (74.4%)	59 (95.2%)	93 (93.0%)	
Hypoxia	56 (20.9%)	2 (22.2%)	43 (25.3%)	4 (12.1%)	7 (12.5%)	0.115
Hypotension	31 (13.2%)	0 (0.0%)	24 (14.8%)	0 (0.0%)	7 (14.3%)	
Glasgow at admission	7.5 ± 4.3	8.0 ± 5.3	4.6 ± 1.9	10.7 ± 1.1	13.6 ± 1.6	<0.001
Major extracranial injury	267 (55.2%)	0 (0.0%)	188 (60.1%)	29 (44.6%)	50 (47.6%	0.01)
Glasgow motor score						<0.001
1	71 (14.7%)	1 (50.0%)	70 (22.6%)	0 (0.0%)	0 (0.0%)	
2	18 (3.7%)	0 (0.0%)	18 (5.8%)	0 (0.0%)	0 (0.0%)	
3	22 (4.6%)	0 (0.0%)	22 (7.1%)	0 (0.0%)	0 (0.0%)	
4	61 (12.6%)	0 (0.0%)	61 (19.7%)	0 (0.0%)	0 (0.0%)	
5	153 (31.7%)	0 (0.0%)	111 (35.8%)	41 (64.1%)	1 (0.9%)	
6	158 (32.7%)	1 (50.0%)	28 (9.0%)	23 (35.9%)	106 (99.1%)	
Prothrombin time (INR)	1.4 ± 0.5	1.4 ± 0.2	1.4 ± 0.6	1.3 ± 0.3	1.3 ± 0.3	0.056
Thromboplastin time partial test	1.2 ± 0.4	1.2 ± 0.3	1.2 ± 0.5	1.1 ± 0.3	1.1 ± 0.3	0.39
Midline brain shift > 5 mm	122 (23.6%)	5 (14.3%)	77 (24.8%)	20 (31.2%)	20 (18.5%)	0.134
Obliteration of basal cisterns	29 (5.6%)	3 (8.6%)	22 (7.1%)	3 (4.7%)	1 (0.9%)	0.092
Sub-arachnoid hemorrhage	222 (42.9%)	10 (28.6%)	147 (47.4%)	29 (45.3%)	36 (33.3%)	0.021
Epidural hemorrhage	407 (78.7%)	28 (80.0%)	253 (81.6%)	45 (70.3%)	81 (75.0%)	0.159
Intracerebral hemorrhage	215 (41.7%)	15 (42.9%)	253 (81.6%)	28 (43.8%)	51 (47.7%)	0.457
Subdural hemorrhage	169 (32.7%)	17 (48.6%)	108 (34.8%)	20 (31.2%)	24 (22.2%)	0.018
Death up to 14 days	118 (22.8%)	8 (22.9%)	81 (26.1%)	11 (17.2%)	18 (16.7%)	0.145
In-hospital mortality	160 (30.9%)	11 (31.4%)	111 (35.8%)	16 (25.0%)	22 (20.4%)	0.017
CCF mortality	19 (3.7%)	1 (3.0%)	15 (4.9%)	2 (3.2%)	1 (0.9%)	0.311

*ICU, intensive care unit; CCF, chronic care facility (Suzano Hospital)*.

### Exploratory Analysis

When evaluating the association between GCS and various outcomes of interest, we found that a worse GCS at admission was significantly associated with an increased risk of mortality at 14 days and in-hospital mortality (*p* < 0.001). However, there was no significant association between age and length of stay at the hospital and in the intensive care unit.

### Model Performance

**Figure 1:**
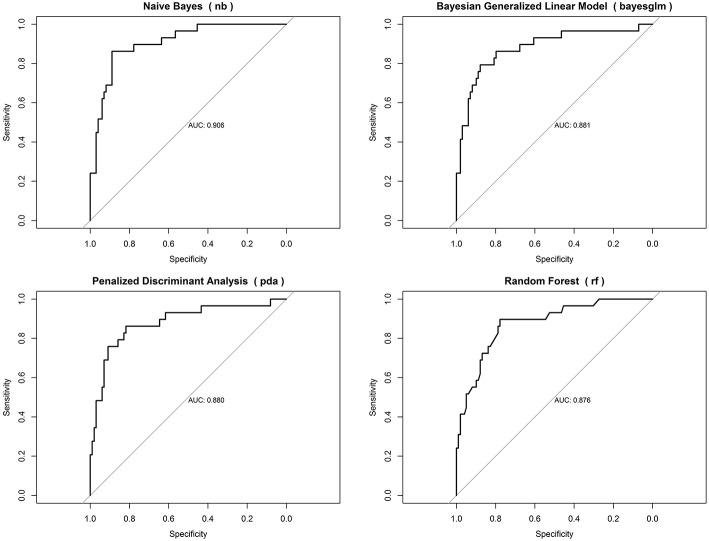
Area under the curve for top-performing models predicting 14 day mortality.

When evaluating models for mortality prediction, we found that naive Bayes had the best predictive performance (area under the curve = 0.906), followed by Bayesian generalized linear model (area under the curve = 0.881), random forest (area under the curve = 0.880), and penalized discriminant analysis (area under the curve = 0.880) (Figure 1).

**Figure 2:**
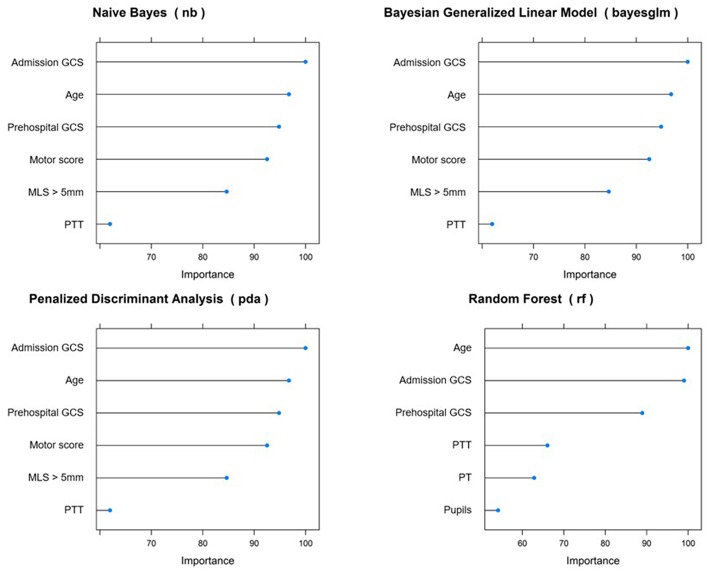
Variable importance across top-performing predictive models for 14 day mortality. GCS, Glasgow Coma Score; MLS, midline shift; PTT, partial thromboplastin time; PT, prothrombin time.

Across all top-performing models, the following variables were consistently considered among the most important: prehospital GCS and GCS at admission, age, and Glasgow motor score (Figure 2).

**Figure 3:**
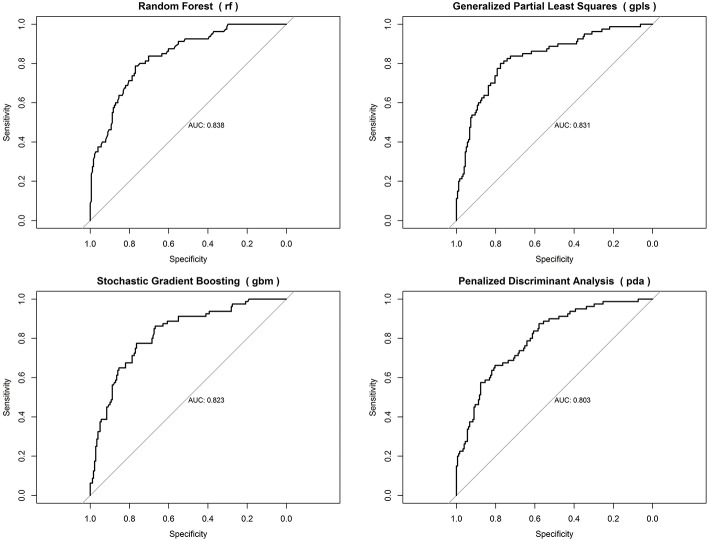
ROC curves for best-performing predictive models for in-hospital mortality.

When predicting in-hospital mortality, random forest was the best performing model (area under the curve = 0.838), closely followed by generalized partial least squares (area under the curve = 0.831), stochastic gradient boosting (area under the curve = 0.823), and penalized discriminant analysis (area under the curve = 0.803) (Figure 3).

**Figure 4:**
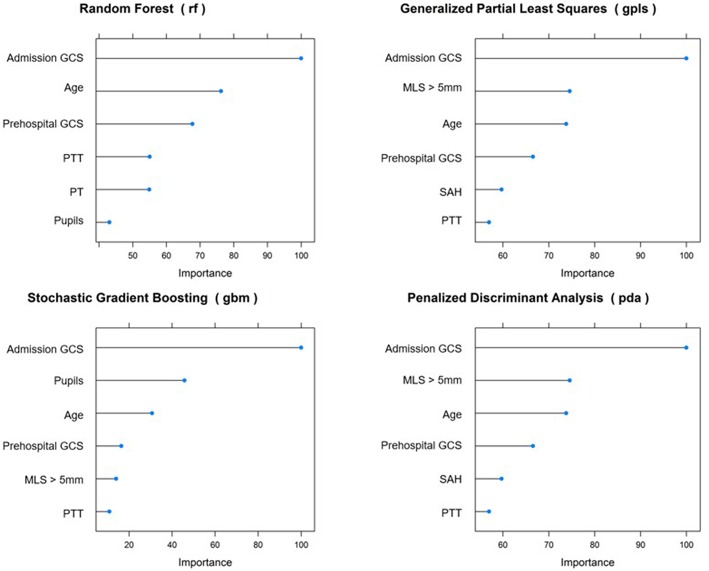
Most important variables predicting in-hospital mortality. GCS, Glasgow Coma Score; MLS, midline shift; PTT, partial thromboplastin time; PT, prothrombin time.

The most important variables predicting in-hospital mortality across all models were GCS at admission, age, prehospital GCS, and thromboplastin time partial test (Figure 4).

## Discussion

The majority of the literature in TBI, including prediction models, comes from developed countries in North America and Europe. However, there are several differences between high-income countries and LMICs that may hinder the external validation of these models including population characteristic, health system, prehospital care, in-hospital care, financial resources, etc. To the best of our knowledge, this is the first study using machine learning to predict mortality and the length of stay after TBI in a LMIC. We found overall mortality at 14 days of 22.8%, with mortality rates increasing proportionally to severity levels. Higher severity scores were also associated with increased rates of epidural, subdural, subarachnoid, and intracerebral hemorrhage levels, as well as with worse GCS scores. Models had a high prediction performance, with the best prediction for overall mortality achieved by naive Bayes (area under the curve = 0.908), with the most significant predictors being GCS at admission, prehospital GCS, age, and pupil reaction. When predicting the length of stay at the intensive care unit, the conditional inference tree model presented the best performance (root mean square error = 1.011), with the most important variable across all models being prehospital GCS. Importantly, our results are equivalent or superior to the best predictive models in the literature reporting on patients in developed countries.

In alignment with our findings, the literature has consistently associated higher mortality rates with lower admission GCS (19, 20). A few studies have also evaluated whether specific sub-components of the GCS could predict mortality, with motor scores equal to or lower than 3 having been associated with higher risk (21). In addition, because the GCS can be measured across the whole care pathway, some authors have sought to investigate at which point the assessment had a stronger association with mortality risk. Previous findings point to the best prediction occurring when combining scores measured at the trauma and admission sites, which is reinforced by our findings (22). Finally, previous publications have frequently predicted GCS themselves by using a range of other clinical characteristics including advanced age and CT findings, which once again points to a possible improvement in their predictive performance if these variables were to be taken into consideration while generating predictive models (23, 24).

Also in consonance with our study, higher mortality has been previously associated with subarachnoid hemorrhage and altered pupil response including bilateral mydriasis, anisocoria, and absence of pupil reflex (19, 24). These findings agree with our current mechanistic understanding of causes leading to death in that subarachnoid hemorrhage may cause vasospasm and subsequent ischemia (25). Specifically, the maximum thickness of the subarachnoid blood has been previously demonstrated to independently predict mortality (26). As expected, previous studies have illustrated that adding a variable indicating sub-arachnoid hemorrhage to predictive models might enhance their predictive accuracy (27).

Concerning the partial thromboplastin time test which was found to be an important predictor of in-hospital mortality, we found that early coagulopathy is associated with a 4-fold increase in mortality among traumatic brain injury patients (28). Also, an increase in partial thromboplastin time of over 1.5 times the standard value was associated with a decrease in median survival times (28), which strongly suggests that this clinical factor should be taken into consideration while evaluating prognosis in LMICs. Other researches have already focused on the relation between partial thromboplastin time test and TBI prognosis. In 2013, Chhabra et al. reinforced the high incidence of coagulopathy following TBI and demonstrated that the presence of coagulopathy is a strong predictor of in-hospital mortality (29). In agreement with these results, a recent article by Yuan et al. revealed that coagulation tests could improve the predictive power of the standard models for in-hospital mortality after TBI (30). The finding of PTT as predictor deserves special attention, especially since the main validated prognostic models, Corticosteroid Randomization After Significant Head Injury (CRASH) and International Mission for Prognosis and Analysis of Clinical Trials in Traumatic Brain Injury (IMPACT), do not consider such variable. It has to be further clarified whether the partial thromboplastin time test can be especially useful in low- and middle-income countries, where investigative tools are scarce. Interestingly, most of the modeling techniques identified the importance of PTT, but did not identify such strong association with prothrombin time (PT) which is somehow hard to explain but should be better analyzed in a future cohort of patients.

Lastly, although the literature on the association between GCS and length of stay in the intensive care unit is scarce, indirect evidence comes from an increase in hyperthermia, which in turn can predict the increased length of stay (31). In contrast with the length of stay in the intensive care unit, there is previous evidence pointing to the severity of traumatic brain injury as a predictor of increases in the overall length of stay (32). Both of these findings corroborate our results.

This paper evaluated several modeling techniques, including logistic regression (the most commonly used in other papers). Even though some studies depict some criticisms regarding their use in medicine, we believe that due to the paucity of studies regarding such methodology, if the investigators can perform several techniques simultaneously (i.e., R packages), this issue should be further investigated (33).

Despite bringing novelty into the literature, our study does have limitations frequently associated with observational designs. First, our diagnoses and outcomes were not validated through observer agreement studies, thus introducing a potential bias. Second, we did not include long-term functional outcomes or self-reported measures of quality of life. In LMICs, the long-term follow-up may be challenging due to difficulties in contacting patients even through mobile phone. Additionally, lack of standardized rehabilitation programs for such patients may contribute for this inadequate follow-up. Due to the inherent features of this LMICs' cohort, collection of variables such as length of coma or duration of post-traumatic amnesia was not possible. Although the endpoint of “14 day mortality” is a short outcome, we do believe it has great importance in patients of LMICs. A couple of the major issues are the pre-hospital care and medical treatment in the acute phase. Therefore, we considered that 14 day mortality should be a strong endpoint to evaluate the treatment in the acute phase indirectly. It may be additionally compared with existing models such as the CRASH trial, which has the same endpoint. Such analysis will be performed in a future study of our group.

Moreover, the self-reported measure constitutes an essential parameter in that they introduce a direct perspective from patients, which is missing when only provider-driven measures are used. This deficiency primarily resulted from the logistical issues related to collecting data in our environment. Additionally, the fact that the sample population includes only patients admitted to ICU may affect generalizability since patients sustaining brain abnormalities could have faced the absence of an ICU bed and had not been transferred. Finally, given that we did not randomly draw out our sample from a larger patient population, its external validity can be questioned. Although future studies should aim at larger and more representative samples, our sample is by no means atypical for its setting, making our conclusions valid for similar populations around the globe. In conclusion, we believe that our model may serve as basis for the development of machine-learning-based algorithms in the prediction of short-term outcomes in LMICs, provided that their population and healthcare system bear a reasonable similarity to ours. Besides the clinical information and assessment of the medical care provided in LMICs, the prediction models may be a useful tool to establish priorities and guide the allocation of limited resources. We also expect that our study might provide the incentive for other centers in LMICs to create similar registries, thus optimizing the prediction of clinical outcomes and improving the quality of care provided to patients with traumatic brain injuries.

## Data Availability Statement

The datasets generated for this study are available on request to the corresponding author.

## Ethics Statement

The studies involving human participants were reviewed and approved by Cappesq–Ethics Committee for Analysis of Research Projects, University of São Paulo Medical School. The patients/participants provided their written informed consent to participate in this study.

## Author Contributions

RA: conception and design, data collection, analysis and interpretation, drafting of the manuscript, reviewing, and final approval of the manuscript. LO and LMi: conception and design, data collection, and reviewing. LMa, AK, and WP: conception and design and reviewing. MN and MS: data collection and reviewing. EB-S-S, AD, and AR: drafting and reviewing of the manuscript. AB-F: reviewing the manuscript. MT: conception and design, drafting, and reviewing of the manuscript.

### Conflict of Interest

The authors declare that the research was conducted in the absence of any commercial or financial relationships that could be construed as a potential conflict of interest.
